# Effect of ultrasound therapy for orthodontic pain control: A pilot randomized clinical trial

**DOI:** 10.6026/973206300220717

**Published:** 2026-02-28

**Authors:** Sakshi Raina, Ashish Kumar Singh

**Affiliations:** 1 Department of Orthodontics and Dentofacial Orthopaedics, MRIIRS Manav Rachna Dental College, Faridabad, Haryana, India

**Keywords:** Orthodontic pain, therapeutic ultrasound, visual analog scale, non-pharmacological analgesia, pilot study

## Abstract

Orthodontic pain has emerged to be the most common side effect to be reported in the course of fixed appliance therapy, with prevalence of more than 70 per cent of patients and may undermine the adherence to treatment. Therefore, it is of interest to controlled pilot trial that tested the analgesic effect of therapeutic ultrasound on pain management in the first stage of alignment. Seven orthodontic patients (15-25 years old) with Class I malocclusion were randomly assigned to the ultrasound (1 MHz, 0.5 W/cm^2^, pulsed mode) or sham treatment during 10 minutes on the second day after the placement of archwires. A visual analog scale (VAS) was used to measuring levels of pain before and after intervention. The ultrasound group recorded statistically significant difference of mean VAS scores of 5.8 + 1.2 to 2.1 + 0.9 (p<0.001), a 63.8% reduction in pain whereas the control group recorded a slight change of 5.6 + 1.1 to 5.2 + 1.0 (p=0.18). No side effects were noticed. Therapeutic ultrasound seems to be a safe, non-invasive and effective modality in the management of orthodontic pain and its use should be applied in larger studies.

## Background:

Pain accompanying orthodontic therapy is the most common and most uncomfortable side effect of the fixed appliance therapy treatment and prevalence rates have been reported to be more than 70% in the initial stages of alignment therapy [1]. The cause of this nociceptive response is the application of mechanical force to periodontal ligaments and leads to the development of complex inflammatory cascades with the release of prostaglandins, cytokines and neuropeptides that sensitize peripheral nerve endings and activate central pain pathways [2]. It is always observed clinically that pain normally starts 4-6 hours after the activation of archwires, peaks at 24-48 hours and slowly disappears in 5-7 days [3]. The severity of this discomfort often causes patients to visit an emergency room and can also cause discontinuation of treatment, which is an important obstacle to positive orthodontic results [4]. The traditional pharmacological treatment is mainly based on non-steroidal anti-inflammatory drugs (NSAIDs), with which, although efficacy was proven, have significant risks such as gastrointestinal ulceration, liver dysfunction and kidney failure when used long-term [5]. Also, there is emerging evidence that NSAIDs could hamper the best orthodontic tooth movement by inhibiting production of prostaglandins required in remodeling of alveolar bone [6]. These constraints have led to exploration of non- pharmacological options such as low-level laser therapy, vibratory devices, transcutaneous electrical nerve stimulation as well as therapeutic ultrasound [7]. Nevertheless, numerous of these modalities either demand active collaboration with the patient, or are associated with high equipment cost and have a weak evidence base to support commonplace clinical practice. Therapeutic ultrasound involves the use of ultrasound high-frequency acoustic vibrations to produce mechanical vibrations and localized thermal effects in deep tissues, with the theoretically proposed mechanisms being the improvements in blood perfusion, decreased inflammatory edema and faster tissue repair [8]. Although ultrasound has shown effectiveness in the management of musculoskeletal diseases as well as temporomandibular joint diseases, its use in orthodontic pain is yet to be fully exhausted [9]. Earlier studies have used non-homogeneous parameters of treatment, small samples and have not been able to use standardized protocols, thus showing contradictory findings [10]. The majority of the research was only on ultrasound without direct comparisons to control conditions with the treatment frequency, intensity and duration being not determined [11]. Further, there is no agreement on time of intervention in comparison to the archwire activation where some of the protocol utilizes treatment instantly and some postpones several hours afterwards [12]. Therefore, it is of interest to determine the effectiveness of therapeutic ultrasound in pain management of orthodontic procedure in the preliminary phase of the initial alignment stage and to provide parameters to be used in further definitive studies on sample size.

## Materials and Methods:

## Study design and setting:

This was a pilot randomized, sham-controlled clinical trial that was done during the year March 2024 to August 2024 in the Department of Orthodontics and Dentofacial Orthopedics, Manav Rachna Dental College, Faridabad. Participants gave written informed consent and parental consent was sought in the case of the participants below the age of 18.

## Participant or sample size and selection:

Since this was a pilot study, a convenient sample of seven patients was formed due to the feasibility factor and pilot trial suggestions in the clinical research. Eligibility screening of consecutive patients who wanted complete orthodontic care was done.

## Inclusion criteria:

Age 15 to 25 years, malocclusion type Class I, with mild to moderate crowding (2-5 mm), need to use non-extraction-based alignment and use 0.014-inch nickel-titanium arch wires as initial alignment wires.

## Exclusion criteria:

The patient had undergone orthodontic treatment, had carious lesions or restorations on the anterior teeth, had an active periodontal disease, had a systemic disease (diabetes mellitus, immunological disorders), was pregnant or had a history of epilepsy, a cardiac pacemaker, or allergy to ultrasound coupling gel.

## Randomization and allocation:

Simple randomization was done by an independent statistician who was not involved in data collection by use of computer generated randomization. The concealment of allocation was done on sequentially numbered, sealed opaque envelopes that were opened upon the enrolment of the patient. Because of the characteristics of the intervention, the clinician treating individuals could not be blinded but the outcome assessors and participants could not be aware of group assignment during the research process.

## Interventions:

Each patient was fitted with pre-adjusted edgewise appliances with 0.022-inch slot MBT prescription brackets bonded with light-cure composite resin (Transbond XT, 3M Unitek). Elastomeric ligatures were used to interact with initial 0.014-inch super-elastic nickel- titanium archwires.

## Ultrasound Therapy Group (n=4):

A therapeutic ultrasound unit (US111, Ultracare Pro) operated in pulsed-mode was used with a spatial average temporal intensity of 0.5 W/cm^2^ and a 20 percent duty cycle. An extraoral sound head of 5 cm^2^ shape was placed over the anterior part of the maxilla and mandible and rotated slowly in circular movement of 10 minutes. Sterile ultrasound coupling gel was to guarantee sufficient acoustical transmission but not to direct contact with brackets and arch wires. Therapy was given after two days of the placement of arch wires as it is when the peak levels of inflammation occur.

## Sham Control Group (n=3):

The patients in the control group had the same procedure and the ultrasound device was turned off upon seeing the initial power-on indication. The transducer was rubbed equally on the target areas and over the target areas the coupling gel was used in keeping the participants blinded.

## Outcome assessment:

The next major result was the pain intensity with the help of a 10-point visual analog scale (VAS) with the range 0 indicating no pain and 10 the worst imaginable pain. At two time points (immediately before treatment and immediately after the 10-minute intervention), patients scored their level of pain on a horizontal 100-mm line. Duration of pain relief, patient acceptance measured using a 5-point Likert scale and adverse events were among the secondary outcomes.

## Statistical analysis:

The analysis of data was done using the version of SPSS 25.0. Since the sample size is small, Shapiro-Wilk test was used to determine normalcy. Mean and standard deviation were used to show the continuous variables. Paired t-test was used in within-group comparisons when data were normally distributed and Wilcoxon signed-rank test when the data were not normally distributed. Independent t-test or Mann-Whitney U respectively was used to assess differences between groups. The statistical significance was defined to be p<0.05. The effect size was computed with the help of Cohen s to determine clinical significance.

## Results:

Seven patients (four females, three males) with mean age 18.4±2.6 years completed the study without attrition. Baseline demographic and clinical characteristics were comparable between groups (Table 1). The ultrasound group comprised four patients (mean age 18.5±2.9 years) and the sham group included three patients (mean age 18.3±2.3 years; p=0.93). Initial crowding severity was similar between groups (ultrasound: 3.2±0.8 mm; sham: 3.0±0.7 mm; p=0.78). Baseline VAS scores were statistically equivalent between groups (ultrasound: 5.8±1.2; sham: 5.6±1.1; p=0.83), confirming comparable pain levels prior to intervention. The ultrasound group demonstrated a statistically significant reduction in mean VAS scores following treatment (5.8±1.2 to 2.1±0.9; p<0.001), representing a 63.8% decrease in pain intensity. In contrast, the sham group exhibited minimal change (5.6±1.1 to 5.2±1.0; p=0.18), with only 7.1% reduction (Table 2). The between-group difference in post-treatment VAS scores was highly significant (mean difference: 3.1; 95% CI: 1.8-4.4; p<0.001). Effect size analysis revealed a large clinical effect (Cohen's d=3.2). Patient acceptance ratings favored ultrasound therapy, with all four patients reporting "very satisfied" or "satisfied" compared to one patient in the sham group (Table 3). No adverse events, tissue damage or bracket debonding occurred during or after interventions. All patients completed the prescribed treatment without reporting discomfort from the procedure itself.

## Discussion:

The current pilot randomized trial proves that the effect of therapeutic ultrasound on orthodontic patients is significant and statistically significant pain reduction during the first crucial phase of alignment. The reduction in VAS scores of 63.8 percent that was immediate after treatment is higher than the minimal clinically important difference of orthodontic pain which has been determined to be around 15 mm on the 100 mm VAS scale [13]. These results present some of the early indications to support the use of ultrasound as a potentially useful non-pharmacological substitute to standard analgesics, especially considering the increasing apprehensions over NSAID-associated problems and the possible negative effect on the mechanics of tooth movement [14]. The pathways of action of ultrasound analgesic effect are probably numerous synergistic mechanisms. The frequency used in this research is 1 MHz which penetrates the body about 3-5 cm which is the equivalent of periodontal ligaments and alveolar bone where the inflammatory mediator concentrates [15]. Pulsed delivery produces cell membrane permeability, stable cavitation and acoustic microstreaming, which removes pain-producing metabolites, including prostaglandin E2 and bradykinin [16]. Also, regulated heating effects increase local tissue temperature 13°C and improve the blood perfusion and speed-up the resolution of the inflammatory cascade triggered by the orthodontic forces [17]. This multimodal act responds to peripheral sensitization and central pain processing with total analgesia as compared to single-mechanism intervention. Transcutaneous electrical nerve stimulation has been shown to produce relatively small effects on pain in some orthodontic pain research, which is due to solely 20-40 percent pain alleviation [18]. The usage of low-level laser therapy has been inconsistent, with the systematic reviews finding mixed outcomes in different studies because of the different parameters used and poor blinding [9]. These modalities are less effective in comparison to the present ultrasound protocol in the 63.8% reduction, indicating the best clinical effect. Moreover, the non-invasive features of ultrasound and the fact that the patient does not have to cooperate with the ultrasound practitioner to facilitate the use of ultrasound improve its practical application in orthodontics with a high workload. The decision to intervene on the second day after archwire placement is a strategic choice, which is dependent on the chronobiology of pain. Maximum levels of inflammatory mediators occur at 24-48 hrs and this is when maximum pain occurs [3]. Ultrasound application at this window provides optimal application to the hyperalgesic period where periodontal tissues were most sensitive. Future research may investigate whether immediate intervention right after an activation or delayed intervention at 48 hours may have different effects and thus define the best therapeutic windows. Clinical translation is supported by the patient acceptance and safety profiles in this trial. No occurrences of adverse events, as well as positive satisfaction scores, portray excellent tolerability. In contrast to the pharmacological methods which have systemic risks, ultrasound has localized effects and no biochemical effects on the bone remodeling mechanisms which are necessary in orthodontic tooth movement [1].

The mobility of current ultrasound machines means that prior barriers of cost and accessibility become less significant and it is possible to incorporate it in normal orthodontic processes. There are a number of constraints that need to be mentioned. The limited sample size of the pilots is a limitation to statistical power and to generalizability, although this was predetermined to conduct a preliminary feasibility check. Lacks of long term follow up after immediate post treatment examination does not allow the analysis of long term analgesic duration. Also, the absence of a no-treatment control (without the sham procedure) could have provided a certain degree of placebo effect though the extent of pain alleviation was significantly greater than the amount of placebo effects in orthodontic pain research [2]. The subjective quality of the VAS assessment, although regarded as the gold standard in pain measurement, presents the possible reporting bias, which can be avoided with objective biomarkers in future studies. This pilot study suggests methodological issues of definitive trials. External validity would be improved by using larger sample sizes that would be recruited across several centers. The objective mechanistic understanding would be offered by incorporation of salivary biomarkers like prostaglandin E2 and substance P. Long-term benefits would be explained with long-term follow-ups looking at the patterns of pain recurrence and its cumulative effects in several appointments. The healthcare policy would be informed by cost- effectiveness studies that present the comparisons between ultrasound and conventional analgesic regimens. The implications involved in the clinic are not limited to pain management. Comfort during early alignment could produce a better adherence to oral health requirements, fewer cancellations and decrease the rates of untimely termination of the treatment. The creation of evidence-based non-pharmacological guidelines is consistent with the current directions of precision medicine that enable providing pain treatment individually and reducing the adverse effects of medications [5]. The 10-minute treatment period was shown to be standardized and unlikely to cause much disruption to the workflow, which implies the possibility of a chair side administration. To sum everything up, therapeutic ultrasound shows great potential in managing orthodontic pain, shows no serious adverse effects and has a very high success rate. These preliminary results serve as the basis to large randomized controlled studies to determine conclusive clinical principles and to streamline treatment variables to integrate them in the orthodontic routine practice.

## Conclusion:

Reduction in the intensity of orthodontic pain of 63.8% at the first stage (alignment) with therapeutic ultrasound was significantly better than with sham therapy. The pilot study has shown that ultrasound is a well-accepted, non-invasive and relatively safe modality in the management of acute orthodontic pain. Thus, we show the development of large-scale randomized controlled trials with extended follow-up and objective bio-marker evaluation to determine conclusive evidence-based ideas on integrating ultrasound in the orthodontic routine management of pain.

## Figures and Tables

**Table 1:** Baseline demographic and clinical characteristics

**Characteristic**	**Ultrasound Group (n=4)**	**Sham Group (n=3)**	**p-value**
Age (years)	18.5±2.9	18.3±2.3	0.93
Female (n, %)	2 (50.0)	2 (66.7)	0.69
Initial crowding (mm)	3.2±0.8	3.0±0.7	0.78
Baseline VAS score	5.8±1.2	5.6±1.1	0.83

**Table 2:** Pain intensity scores and reduction

**Group**	**Baseline VAS**	**Post-treatment VAS**	**Mean Reduction**	**% Reduction**	**p-value**
Ultrasound (n=4)	5.8±1.2	2.1±0.9	3.7±0.8	63.8±12.4	<0.001
Sham (n=3)	5.6±1.1	5.2±1.0	0.4±0.5	7.1±8.9	0.18
Between-group p-value	0.83	<0.001	<0.001	<0.001	-

**Table 3:** Patient acceptance and safety outcomes

**Outcome Measure**	**Ultrasound Group (n=4)**	**Sham Group (n=3)**	**p-value**
Very satisfied (n)	3	0	0.04
Satisfied (n)	1	1	-
Neutral (n)	0	1	-
Dissatisfied (n)	0	1	-
Adverse events (n)	0	0	-
